# Programmable hydraulic resistor for microfluidic chips using electrogate arrays

**DOI:** 10.1038/s41598-019-53885-w

**Published:** 2019-11-21

**Authors:** Marie L. Salva, Yuksel Temiz, Marco Rocca, Yulieth C. Arango, Christof M. Niemeyer, Emmanuel Delamarche

**Affiliations:** 1 IBM Research – Zurich, Säumerstrasse 4, 8803 Rüschlikon, Switzerland; 2 Karlsruhe Institute of Technology (KIT) – Institute for Biological Interfaces (IBG-1), Hermann-von-Helmholtz-Platz 1, 76344 Eggenstein-Leopoldshafen, Germany

**Keywords:** Microfluidics, Characterization and analytical techniques

## Abstract

Flow rates play an important role in microfluidic devices because they affect the transport of chemicals and determine where and when (bio)chemical reactions occur in these devices. Flow rates can conveniently be determined using external peripherals in active microfluidics. However, setting specific flow rates in passive microfluidics is a significant challenge because they are encoded on a design and fabrication level, leaving little freedom to users for adjusting flow rates for specific applications. Here, we present a programmable hydraulic resistor where an array of “electrogates” routes an incoming liquid through a set of resistors to modulate flow rates in microfluidic chips post-fabrication. This approach combines a battery-powered peripheral device with passive capillary-driven microfluidic chips for advanced flow rate control and measurement. We specifically show a programmable hydraulic resistor composed of 7 parallel resistors and 14 electrogates. A peripheral and smartphone application allow a user to activate selected electrogates and resistors, providing 127 (2^7^-1) flow resistance combinations with values spanning on a 500 fold range. The electrogates feature a capillary pinning site (i.e. trench across the flow path) to stop a solution and an electrode, which can be activated in a few ms using a 3 V bias to resume flow based on electrowetting. The hydraulic resistor and microfluidic chip shown here enable flow rates from ~0.09 nL.s^−1^ up to ~5.66 nL.s^−1^ with the resistor occupying a footprint of only 15.8 mm^2^ on a 1 × 2 cm^2^ microfluidic chip fabricated in silicon. We illustrate how a programmable hydraulic resistor can be used to set flow rate conditions for laminar co-flow of 2 liquids and the enzymatic conversion of a substrate by stationary enzymes (alkaline phosphatase) downstream of the programmable hydraulic resistor.

## Introduction

Flow of liquids at the microscale is peculiar and affect most of the processes occurring in microfluidic devices. They determine the interplay between convection and diffusion, how much sample and reagents pass and may be consumed in a device, shear stress on objects such as cells in microchannels, and affect mixing and dissolution patterns of reagents^1,2^. For these reasons, great care has been devoted to controlling the flow of liquids in microfluidics using active and passive systems.

Active flow displacement relies on mechanical parts such as pumps or centrifugal platforms that apply a specific pressure or force to a liquid to achieve specific flow rates in a device. Valves can also be used to modulate flow rates by varying locally the dimensions and hydraulic resistance of a flow path^3–5^. Active flow control therefore necessitates peripherals^6,7^, an energy source^6^, a controller and user interface^7^, and sometimes complex design and fabrication strategies when pumps or valves are integrated to a device^6,8,9^. Flow or pressure sensors might be needed for feedback control and user interventions may lead to mistakes and imprecisions. Active flow control provides flexibility for users but tend to be more expensive and less user-friendly than devices based on passive flow^6,10^.

Setting flow rates in passive microfluidics is typically done on a design and fabrication level by tuning the hydraulic resistance and/or wetting properties of flow paths appropriately^11^. Alternatively, different materials or capillary pumps can be connected serially to change flow rates after a specific volume of the sample has passed in the device^12^. Microfluidic devices are typically microfabricated, replicated using injection molding^13^ or hot embossing^14^, or even produced using 3D printing^15,16^. While such fabrication techniques are amenable to mass manufacturing of microfluidic chips with high accuracy and resolution, specific microfluidic chips must be designed and fabricated with an anticipation of the flow rates that a final device should have. Despite progress done on timing and delaying flow using microfluidic elements involving capillary valves^17^, responsive materials^18,19^, or smart coatings^20,21^, a simple method for selecting a specific flow in a microfluidic device post-fabrication that is based on a simple user intervention is still missing.

Here, we combine capillary-driven microfluidics with battery-powered flow elements to provide a flexible method to control and tune flow rates post-fabrication. This work is based on a concept called “electrogates”, which we previously developed for stop-and-go control of flow of liquids in capillary-driven microfluidic chips^22^. Electrogates combine electrowetting^23–26^ with capillary pinning^27,28^ to stop temporarily a liquid filling a wettable microchannel. Electrogates can be activated using a peripheral and a smartphone application. The programmable hydraulic resistor is realized by coupling electrogates to a set of parallel hydraulic resistors. Activating electrogates upstream and downstream of resistors defines which resistors contribute to the flow path and hydraulic resistance of the entire array. This solution is simple, efficient and does not involve mechanical structures; it instead uses a small form factor peripheral to energize electrogates. With only a few resistors in a programmable array, many combinations of flow resistances can be achieved. We detail the design and fabrication of this concept and illustrate it with experiments involving laminar flows and enzymatic assays.

## Results

### Principle

**Figure 1:**
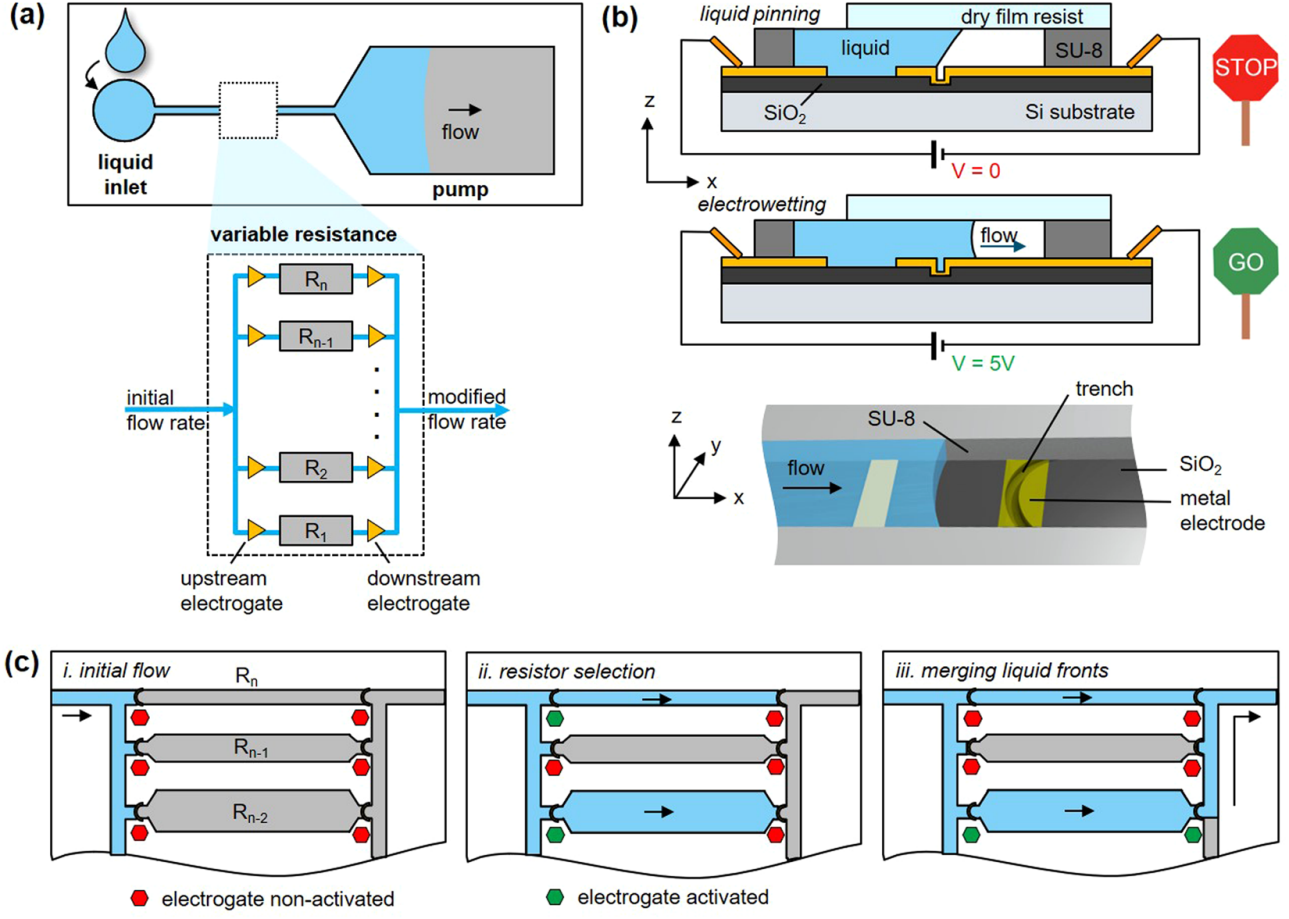
Programmable hydraulic resistor implemented in a capillary-driven microfluidic chip and using electrogates. (**a)** A programmable hydraulic resistor composed of *n* resistors and *2n* electrogates is integrated into a capillary-driven microfluidic chip to modulate the flow rate of a solution passing in the chip. **(b)** An electrogate comprises a geometric discontinuity (e.g. trench), where an incoming liquid gets pinned, and electrodes to which a potential can be applied between the liquid and the trench to induce an electrowetting effect allowing capillary flow of the liquid to resume. **(c)** Individual resistors in the array contribute to the flow path when their electrogates are activated (green sign). All resistors are different and R_1_ < R_2_ < … < R_n_. When several resistances are selected, only the downstream electrogate, which is the furthest away from the output channel is activated to let the liquid pass and connect with menisci momentarily pinned at the non-activated downstream electrogates.

The principle of a programmable hydraulic resistor using electrogate arrays is shown in Fig. 1. The programmable resistor array is part of the flow path of a capillary-driven microfluidic chip and comprises *n* (*n* ≥ 2) parallel resistors (R_1_ < R_2_ < … < R_n_) and 2*n* electrogates, with one electrogate upstream and the other one downstream of each resistor (Fig. 1a). An electrogate combines a geometric discontinuity of the flow path (e.g. a trench) to pin an incoming liquid with a nearby electrode, which can be biased with a low voltage (<5 V) to induce an electrowetting effect to resume flow (Fig. 1b). In a programmable hydraulic resistor using electrogate arrays, an incoming liquid can only pass through individual resistors, which have upstream electrogates activated (Fig. 1c(i)). In the simplest case, a single resistor is used to modulate the flow rate in the microfluidic chip. The downstream electrogate of this resistor can be activated as soon as the liquid reaches it to let the liquid flow through the selected resistor and proceed to the rest of the flow path. If multiple resistors are selected, their corresponding upstream electrogates can be all activated to fill the resistors until the downstream electrogate (Fig. 1c(ii)). Then, only the resistor with the lowest hydraulic resistance is activated to let the liquid pass and connect with the menisci pinned at the other filled resistors (Fig. 1c(iii)). Here, only one electrogate is activated but the pinning sites of the other electrogates are useful and prevent premature exit of the liquids to the connecting channel. Specifically, the output channel connecting the resistors is slightly deeper (16.9 μm) than the resistors (15 μm) to form a capillary valve^29^: no liquid in a filled resistor exits prematurely, which is important to avoid the formation of air bubbles. Having *n* resistors results in a combination of 2^*n*^ − 1 possible resistance values of an array in a single microfluidic chip.

### Design of a programmable hydraulic resistor using electrogates

**Figure 2:**
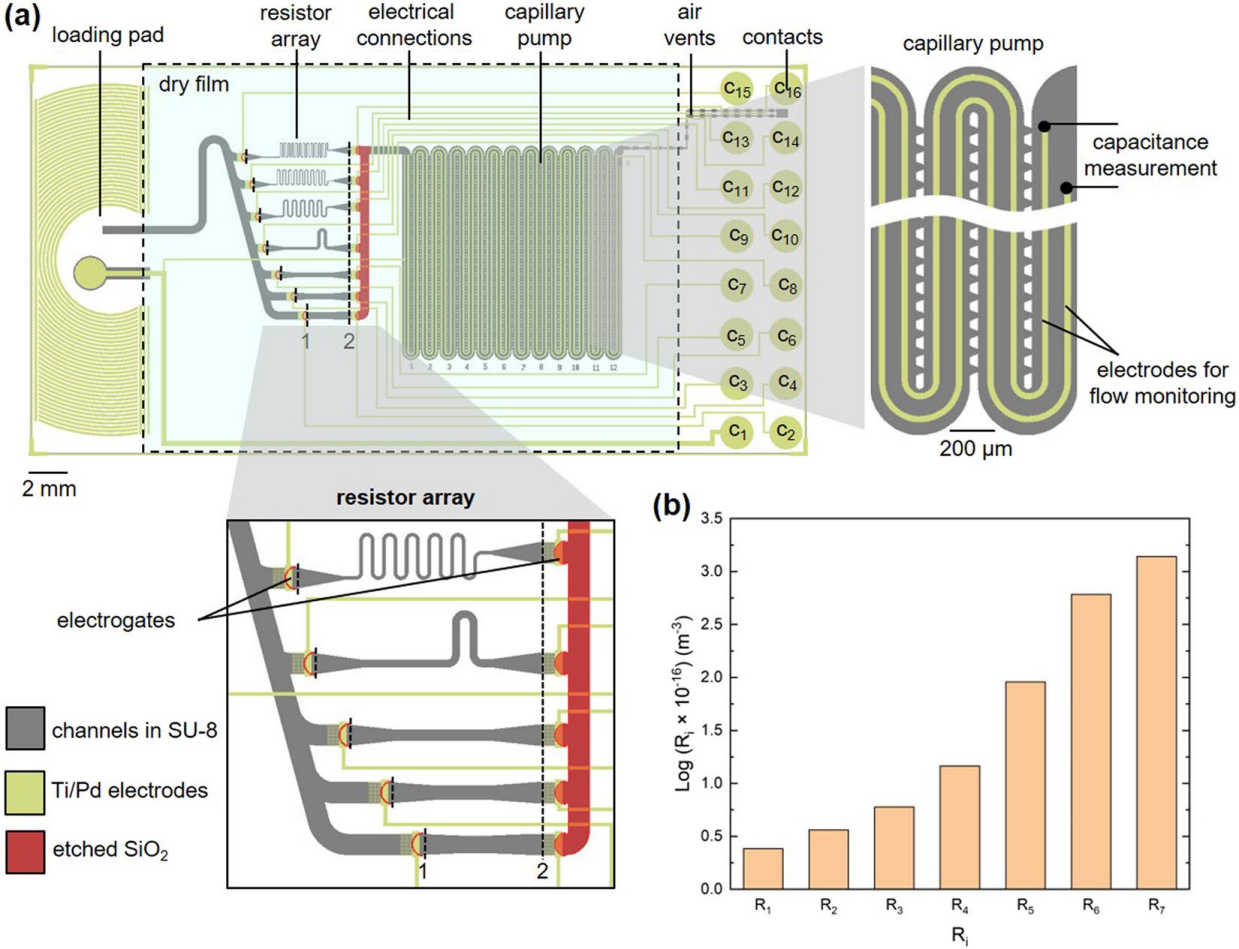
Layout illustrating the integration of a programmable hydraulic resistor in a functional microfluidic chip and theoretical prediction of the flow resistance along flow paths involving only one resistor from the array. (**a)** The microfluidic chip includes a loading pad, a resistor array, a capillary pump, air vents, and 16 contacts for activating specific electrogates and monitoring flow in the microfluidic chip. The chip is sealed between the loading pad and the vents using a laminated dry film resist. The resistor array shown here comprises 7 individual resistors and 14 electrogates, and is serviced by a common microchannel (15-μm-deep, patterned in SU-8). Streams of liquid exiting individual resistors are merged using a deeper channel (16.9 μm: 15 μm in SU-8 and 1.9 μm etched in a SiO_2_ layer). Flow in the capillary pump is monitored by following the evolution of the capacitance across two parallel electrodes. **(b)** Graph showing the calculated flow resistance of individual resistors, i.e. flow path between (1) and (2).

Figure 2a shows the layout and key functions of a capillary-driven microfluidic chip. The chip includes a loading pad on which a liquid is pipetted, the resistor array, a capillary pump, electrical connections, air vents and 16 contacts. A transparent dry film resist seals the flow path located between the loading pad and the vents. The capillary pump has two parallel electrodes (right inset in Fig. 2a), which can be used to monitor flow rates using capacitance measurements^30^. Contacts are used to bias the liquid pipetted to the loading pad (contact 1), electrogates (contacts 2 to 7 and 9 to 16) or electrodes for monitoring flow (contacts 1 and 8) using standard Pogo pins. The design is flexible and the role of each contact can be programmed using the peripheral device. The resistor array shown in Fig. 2 represents 7.9% of the total area of the microfluidic chip and 16.4% of the volume of the capillary pump. A portion of the resistor array is detailed in the inset in Fig. 2a. We imposed conditions for the design of the individual resistors such as keeping the same depth for all resistors and not having any elements smaller than 10 μm to keep the fabrication of the resistors easy, avoid potential clogging issues by particulates in liquids, and have the possibility to fabricate such arrays using replication techniques. The key parameter determining the hydraulic resistance of each resistor is its width (see below); the geometry of electrogates before and after each resistor is kept identical. The microchannel between the loading pad and each resistor is oblique to minimize the footprint of the array. Figure 2b shows the calculated resistance of each resistor in the array between points 1 and 2. A factor of at least 500 is easily achieved between the highest and lowest resistances by just changing the width of the resistor and using a meandering shape to accumulate hydraulic resistance along the flow path. Supplementary Fig. S1 details the layout, dimensions and resulting hydraulic resistances reported in the graph as well as the depths of the hydraulic resistances and connecting channels. The resistance of rectangular portions of the resistors were calculated using the simplified Eq. (1) for a rectangular microchannel (*d* < *w*)^31,32^:

1 $$R=\frac{12L}{(1-0.63\frac{d}{w}){d}^{3}\,w}$$

where *L*, *d*, *w*, are respectively the length, depth and width of the microchannel. This expression does not apply to portions of a resistor where *w* varies. Therefore, the resistance of trapezoidal portions (e.g. between 1 and 3 or 4 and 2 in Fig. S1) was calculated using equations detailed in Supplementary Fig. S2. Here, using a Pogo pin layout/interface having 16 electrical contacts, we were able to design empirically 7 resistors, which necessitated 14 electrogates and contacts. If a particular range of resistance needs to be covered using *n* resistors, and if these resistances should be equally distributed, the resistance of each resistor can be derived according to (2) and (3):

*for n* = 2

2 $$\{\begin{array}{rcl}{R}_{2} & = & {R}_{tot\_max}\\ {R}_{1} & = & \frac{{R}_{tot\_min}{R}_{2}}{{R}_{2}-{R}_{tot\_min}}\end{array}$$

*for n* ≥ 3

3 $$\{{R}_{1}=\begin{array}{c}{R}_{n}={R}_{tot\_max}\\ \frac{{R}_{tot\_min}{\prod }_{i=2}^{n}\,{R}_{i}}{\,({\prod }_{i=2}^{n}\,{R}_{i})-{R}_{tot\_min}{R}_{n}({\sum }_{i=2}^{n-1}\,{\prod }_{j=2;\,j\ne i}^{n-1}\,{R}_{j})-{R}_{tot\_min}{\prod }_{i=2}^{n-1}\,{R}_{i}}\\ {R}_{i+1}-{R}_{i}={R}_{i}-{R}_{i-1},\,\,\,for\,i\,\in \,[2;\,n-1]\end{array}$$

where R_tot_min_ and R_tot_max_ are the desired minimum and maximum resistances of the array, respectively.

### Capillary-driven microfluidic chip having a programmable resistor array

**Figure 3:**
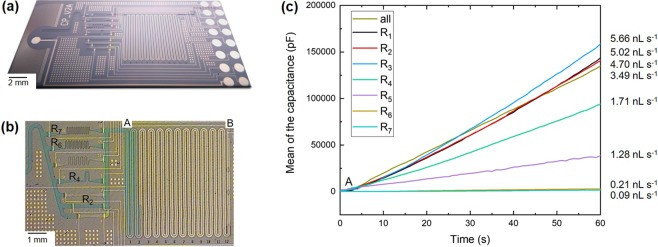
Implementation of a programmable resistor array on a Si microfluidic chip and characterization of the resulting flow rates. **(a)** Photograph of a microfluidic chip fabricated using 3 masks and having a 15-μm-deep flow path. **(b**) Optical micrograph showing PBS containing a blue dye flowing through 4 activated resistors (R_2_, R_4_, R_6_, R_7_) and starting to fill a capillary pump. The flow rate is determined by monitoring the state of filling of the capillary pump between the sections (A) and (B). **(c)** Graphs showing the evolution of the capacitance measured across parallel electrodes patterned in the capillary pump as a function of time for all or a single activated resistor. The graphs are an average from experiments done in triplicate. A corresponding figure with all error bars is provided in Supplementary Fig. S6.

Figure 3 shows a capillary-driven microfluidic chip in Si including a programmable hydraulic resistor, which was used to displace small volumes of PBS at various flow rates. The chip shown in Fig. 3a is ~1 cm × 2 cm in area, 26 such chips can be fabricated on a single 4-inch wafer, and its fabrication process is detailed in Supplementary Fig. S3. Briefly, 3 photolithographic steps are sequentially used to (i) define the trench of electrogates and make deeper the microchannel connecting flows after the array (etched SiO_2_, shown in Fig. 2a in red), (ii) pattern the electrodes for electrogates and flow monitoring (Ti/Pd), and (iii) define the flow path (SU-8). Figure 3b shows an example where 4 resistors (R_2_, R_4_, R_6_, R_7_) were selected to let a solution of PBS containing a blue dye pass the array. The activation was done using a custom peripheral and an application written for Android, Supplementary Fig. S4. Specifically, the chip is inserted on one side of the peripheral and contacted using Pogo pins. A Bluetooth^®^ module, an Arduino microcontroller, and a digital-to-analog converter are used to bias electrogates with a ~3 V during ~800 ms. The energy needed to activate an electrogate is ~1.2 mJ and using a standard CR2032 battery, the peripheral should be able to actuate selected electrogates (once), monitor flow and communicate with a smartphone for at least 90 min as detailed in Supplementary Fig. S5. The application has a user interface for setting electrogate parameters (electrogate selection, voltage to apply and its duration) and for monitoring flow in real time where contacts (ground and signal) can be selected in any combination. Videos and/or images of a meniscus filling capillary pumps were recorded while monitoring the increase of the capacitance between the 2 parallel electrodes patterned in the capillary pump. The steady increase of capacitance corresponds to an increasing wetted area between the parallel electrodes, which allows the flow rate to be determined with an accuracy of ~1 nL.s^−1^ ^30^. In Fig. 3c, each curve represents the average of 3 experiments (see also Supplementary Fig. S6 where error bars are depicted). In this graph, the capacitance is measured for separate experiments where only one or all resistors were activated. As anticipated from the design, resistors having narrow and long microchannels lead to lower flow rates and, conversely, resistors shorter and wider microchannels support stronger flow rates. Flow rates from sub-nL.s^−1^ up to a few nL.s^−1^ are achieved. The capillary pressure generated by the numerous parallel, wettable microstructures in the pump is of the order of −5 to −6 kPa^11^ and most of the hydraulic resistance in the flow path is contributed to by structures upstream of the pump. This helps ensuring a steady flow rate when a liquid gradually fills the pump. Typically, samples or liquids in the form a 5 μL are added to a loading pad starting the flow path. The hydrostatic pressure of such a droplet (~1.3-mm-high) represents ~13 Pa and can be neglected in comparison to the pressure generated by the capillary pump. This is also the case for the Laplace pressure of the droplet, which for an aqueous solution should be of the order of 109 Pa. To place flow rates demonstrated here in perspective, a sample having a volume of just 1 μL can be passed in the microfluidic chip for more than 15 min using a large hydraulic resistance. This is particularly beneficial for bio-analytical applications involving enzyme-substrate or receptor-ligand interactions where sensitivity usually correlates with slower flows and the renewal of analytes in areas nearby amplifying enzymes or receptors^33^. This can be very useful for assays involving biomarkers that can be present at low or high concentration, depending on the clinical application^34^. Specifically, the programmable resistor array can be used to vary the dynamic range of a given microfluidic chip device. Another use of such an array can be to adjust the flow rate of a device in order to accommodate for batch-to-batch manufacturing variability of a diagnostic device.

### Laminar flows and enzymatic assays using a programmable resistor array

**Figure 4:**
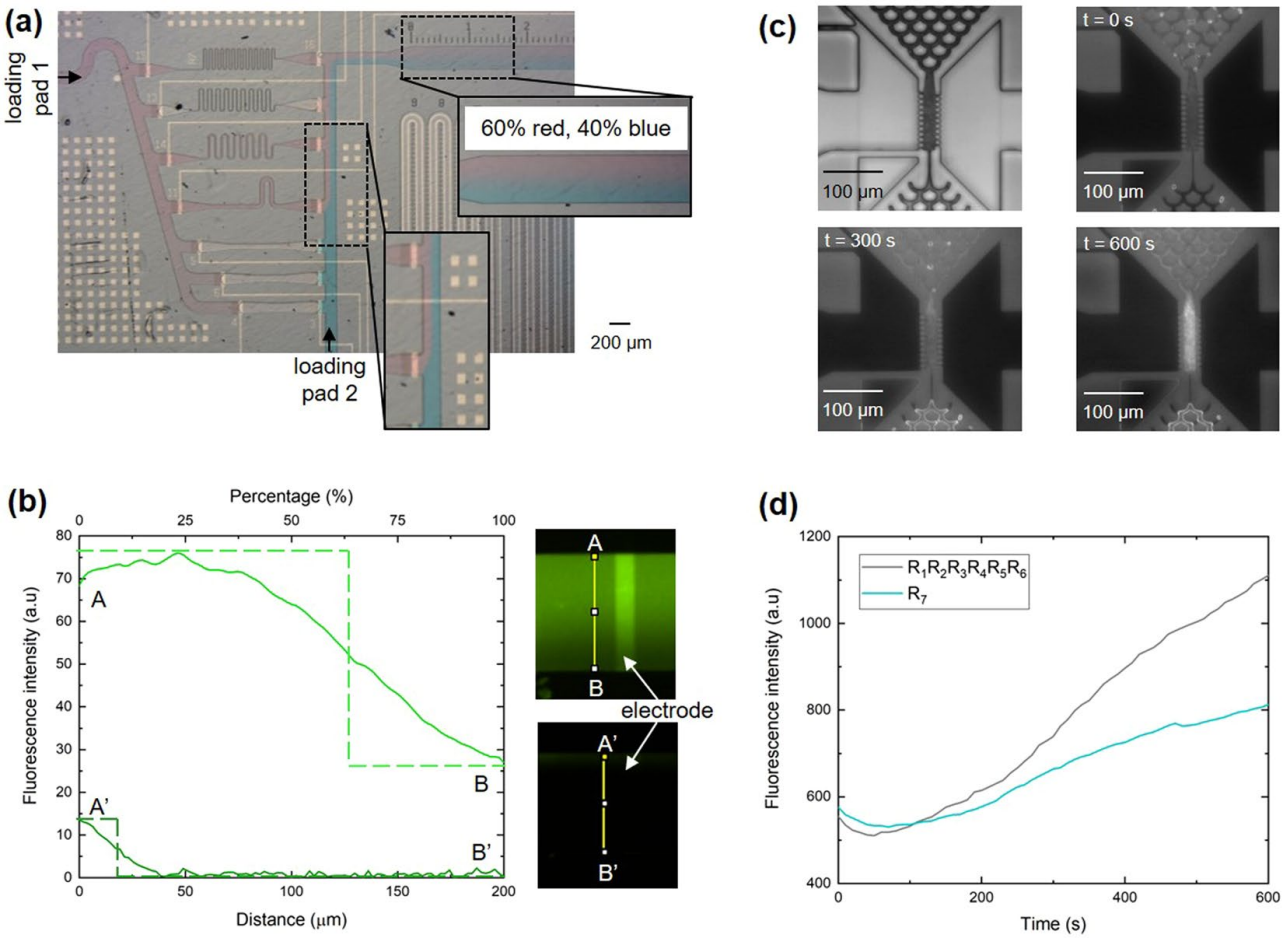
Laminar flow and enzymatic assay using a programmable resistor array. (**a)** Optical micrograph showing the formation of two laminar streams by merging a PBS solution passing through a programmable resistor array (red) with a PBS solution (blue) flowing directly from a second loading pad. **(b)** Graphs showing the intensity profile and the percentage of fluorescent dye in the liquid flowing through the resistor array as a function of the position along the width of the microchannel for all activated resistors (A to B) or for only the highest resistance activated (A’ to B’). Dashed lines represent the step function of each intensity profile. **(c)** Optical microscope images showing the bead lane of a sealed microfluidic chip containing 5 µm beads functionalized with alkaline phosphatase, before flowing a solution of ELF-97 through it (top left, bright field, objective 20×), when time lapse recording started (top right, UV-2A light source and corresponding fluorescence filter, objective 20×), and at a later time. The observed fluorescence signal results from the dephosphorylation of ELF-97 leading to a fluorescent precipitate. **(d)** Graph showing the evolution of the fluorescence intensity related to the enzymatic assay as a function of time and for one (R_7_, highest resistance) or a subset (R_1_, R_2_, R_3_, R_4_, R_5_, R_6_) of activated resistors.

Beyond ligand-receptor assays and diagnostic applications, flow rates also play a fundamental role in chemistry at the microscale^35^, enzymatic processes^36^, DNA sequencing^37^, mixing processes^24,38,39^, and experiments involving laminar flows^40,41^. For this reason, we also illustrate how a programmable hydraulic resistor can be used to modulate laminar flows and enzymatic assays. Laminar flow of liquids in microfluidics has been used to address fundamental questions in developmental biology^41,42^, fabricate structures inside microfluidics^43^, probe chemical-cell interactions using sub-population of cells^44,45^, and detect analytes^46–48^. A microfluidic chip for experiments involving laminar flow of 2 different liquids is shown in Supplementary Fig. S7. This chip includes 2 loading pads, a resistor array, an observation channel to monitor the distribution of both liquids after the array, electrical connections and contacts, and a capillary pump. Three electrogates have been added to the capillary pump to provide the opportunity to stop and resume flow to observe the diffusion of chemical species at the interface between the liquids, if desired. Figure 4a illustrates a laminar flow formed by two colored solutions and where the flow rate of the solution in red was modulated by activating 3 resistors (R_4_, R_5_ and R_7_). In this example, the flow resistance of the flow path of the red liquid is calculated to be 1.91 × 10^17^ m^−3^ compared to 3.25 × 10^17^ m^−3^ for the blue liquid. We therefore expect a ratio of 0.63/0.37 for the red and blue liquids and observed a ratio of 0.6/0.4 in the observation channel. Solutions containing fluorescent species can as well be employed to visualize laminar co-flows. In Fig. 4b., a solution containing fluorescein was pipetted on loading pad 1 and passed the programmable hydraulic resistor with all the resistors activated (lowest hydraulic resistance) or with only R_7_ activated (highest hydraulic resistance); a buffer without any fluorescent molecule pipetted on loading pad 2 provided the second liquid for realizing a co-flow. The contribution of each solution to the flow in the observation channel can be assessed by taking the experimental value of the fluorescence at the intersection between the curve *f*(*x*) of the intensity profile across the width of the microchannel and a step function ${\chi }_{(f(x))}$ defined by (4):

4 $${\chi }_{(f(x))}=\{\begin{array}{ll}{\max }\,f(x), & f(x) > \,{\min }\,f(x)+\,\frac{{\max }\,f(x)-\,{\min }\,f(x)}{2}\,\\ {\min }\,f(x), & f(x) < \,{\min }\,f(x)+\frac{{\max }\,f(x)-\,{\min }\,f(x)}{2}\end{array}$$

where ${\rm{\max }}\,f(x)$ and ${\rm{\min }}\,f(x)$ are the maximum and minimum of the fluorescence intensity measured across the width of the microchannel, respectively^49^. With this method we found a ratio of 0.65/0.35 when all resistors are activated (measurement from A to B) and a ratio of 0.09/0.91 when R_7_ is activated (measurement from A’ to B’). Here, the input channel of loading pad 2 is sequentially connecting the individual outputs of each resistor (see Supplementary Fig. S7a), which adds a complex diffusion pattern of the dye across both liquids. If needed, the liquid from loading pad 2 can be directly connected to the output of the array instead of being combined with the connecting channel of the array. This would create a simpler flow pattern and more distinguishable liquids interface, which can be useful if modelling the diffusion of fluorescent molecules in the co-flow is critical as in the case of diffusion-based sensors^49–51^.

We also illustrate how an enzymatic assay can be carried out using self-assembled microbeads functionalized with an enzyme, alkaline phosphatase, and using different flow rates for supplying an enzymatic substrate, fluorogenic ELF-97. Alkaline phosphatase is commonly used in biological assays^52–54^. It can dephosphorylate ELF-97, resulting in the formation of a strongly fluorescent precipitate that has a significant Stokes shift (λ_exc_ = 345 nm, λ_em_ = 530 nm) and can be detected using fluorescence microscopy. The activity of alkaline phosphatase immobilized on 5 μm PMMA beads was verified “off-chip” (see Supplementary Fig. S8) and subsequently “on-chip” using simplified microfluidic chips (see Supplementary Fig. S9). These simpler chips consisted only in one layer of SU-8 to define the flow path and did not have a programmable hydraulic resistor and electrogates. The hydraulic resistance of the flow path, hence, mimicked only one specific resistance of the programmable hydraulic resistor. To verify “on-chip” the enzymatic activity of alkaline phosphatase by dephosphorylation of ELF-97, microbeads functionalized with alkaline phosphatase were introduced in a structure named bead lane using a dedicated loading pad^55^, and a solution of ELF-97 was pipetted into the loading pad of the microfluidic chip. Measurement of the fluorescent signal intensity resulting from the formation of the precipitating product in the bead lane of simplified microfluidic chips showed a faster signal increase at a high flow rate (all resistors activated, i.e. low hydraulic resistance). Subsequently, the enzymatic reaction was determined with microfluidic chips including a programmable hydraulic resistor (see Supplementary Fig. S10). Figure 4c shows microscope images of the bead lane containing 5 μm beads functionalized with alkaline phosphatase, before flowing ELF-97 across it (image in bright field), and during conversion of ELF-97 (fluorescence images). The accumulation of enzymatic products led to progressively increasing fluorescence on the beads and depended on the resistance of the programmable hydraulic resistor (Fig. 4d). When R_7_ only was activated (highest hydraulic resistance, lowest flow rate) the fluorescence intensity reached ~800 a.u. in 600 s, whereas activating R_1_, R_2_, R_3_, R_4_, R_5_, and R_6_ increased the flow and transport of ELF-97, thereby resulting in typical fluorescence signals of ~1100 a.u. after 600 s. Therefore, the results provide a clear demonstration of how a programmable hydraulic resistor can be used to vary the speed of a mass transfer limited reaction inside a microfluidic device^33^.

## Discussion

We demonstrated how a programmable hydraulic resistor for microfluidic chips using electrogate arrays can be designed and implemented in a capillary-driven microfluidic chip to change the flow properties of such chips post-fabrication. We specifically designed a programmable hydraulic resistor composed of 7 different parallel resistors and 14 electrogates. The fabrication involved 3 photolithography masks and a few standard microfabrication steps. The flow path of the liquid was controlled by activating specific electrogates using a custom-made peripheral and a smartphone application. With such a programmable hydraulic resistor, we were able to modulate the flow rate in a capillary-driven microfluidic chip by a factor of at least 50. This is particularly interesting for point-of-care diagnostic applications, where the duration of an analysis depends on the required sensitivity and dynamic range, and typically assay times range from a few minutes up to an hour.

Like many other techniques, the concept we show here has a few limitations. Firstly, we demonstrated programmable hydraulic resistor arrays using capillary-driven microfluidic chips fabricated in a multi-user prototyping cleanroom using manual SU-8 deposition and DFR lamination steps. Such a fabrication can lead to slight variations in the height of channels, the creation of random defects, and possible photoresist residues on the flow monitoring electrodes, all of which can contribute to variation in the flow rate and the capacitance measurements. However, this issue can be addressed using controlled and calibrated processes using automated tools. Secondly, the capacitance measurements (e.g. Supplementary Fig. S6) show some variation particularly for low resistor values (i.e. high flow rates). We used a long electrode pair covering the whole capillary pump to continuously monitor the filling of the pump, which results in a large double-layer capacitance. In this case, the electronic peripheral cannot charge fast enough to match the fast liquid flow. This can be addressed by patterning a shorter electrode pair passing at the middle of the pump (i.e. monitoring the flow at specific locations) and/or adding an auto-ranging feature to the electronics to adapt the rate of capacitance measurements.

In conclusion, modeling the hydraulic resistance of flow paths can be used to design chips and predict flow properties to reduce the complexity of the array by including a few resistors having resistances differing by several orders of magnitudes. Our approach is not limited to modulating the flow rate of a single liquid passing in a chip. It can also be used to affect biochemical reactions occurring in a microfluidic chip and to control the spatial extension of laminar streams of solutions. Furthermore, on larger microfluidic chips and using more contacts, it should be possible, by implementing a few programmable hydraulic resistors distributed at strategic locations, to realize multiple functions, such as mixing, reagent reconstitution, separation or capture of analytes, perfusion and cell stimulation, each with specific and optimal flow conditions.

## Methods

### Chemicals and biological chemicals

All reagents were purchased from Sigma-Aldrich unless otherwise indicated. PBS was purchased from Biowest and filtered using a 0.45 μm filter (Merck Millipore Ltd.). Colored solutions were prepared by dissolving erioglaucine disodium salt (5 mg/mL) and amaranth (5 mg/mL) in PBS to prepare blue and red solutions, respectively. Fluorescein was dissolved in PBS (1:10, w:v) containing 0.05% (v:v) Tween^®^ 20. 5 μm streptavidin-coated PMMA microbeads (1% solid content) were purchased from PolyAn (Berlin, Germany). ELF^TM^ 97 phosphatase substrate was from ThermoFisher (Waltham, MA, USA). Biotinylated alkaline phosphatase was purchased from Rockland (Plymouth, MA, USA). All preparations were made and used at room temperature.

### Functionalization of microbeads

To exchange the storage buffer of the microbeads to PBS, 5 μL of streptavidin coated microbeads were dissolved in 95 μL PBS and purified 3 times by centrifugation. To this end, the solution was centrifuged 3 times at 1500 × g for 3 min (Centrifuge 5415R from Eppendorf, Hamburg, Germany), the supernatant was removed, and the beads were resuspended in a total volume of 100 μL PBS. After the final centrifugation step, microbeads were resuspended in a total volume of 23.7 μL. Biotinylated alkaline phosphatase was attached to streptavidin coated beads by adding 6.22 μL of biotinylated alkaline phosphatase (6.7 μM stock) giving a 10× excess of biotin binding sites. The resulting solution was incubated for 60 min at room temperature on an orbital shaker (600 rpm). Then, the microbead solution was diluted to a total volume of 100 μL in PBS. Unbound biotinylated alkaline phosphatase was removed with 2 rounds of centrifugation at 1500 × g for 3 min. After round 1, the supernatant was removed, and functionalized beads were resuspended in PBS to a total volume of 100 μL. After round 2, functionalized microbeads were resuspended in 10 μL of PBS.

### Microfluidic chips fabrication

Microfluidic chips were fabricated on a 4-inch silicon wafer having a 3 μm thermally-grown SiO_2_ layer. Trenches for liquid pinning sites (electrogates, connecting channel) were formed by etching the oxide layer to a depth of 1.9 μm using an inductively coupled plasma (ICP2 Plasma ProSystem 100, Oxford Instrument) for 7 min. Electrodes and metal contacts (80-nm-thick Pd layer on top of a 5 nm Ti adhesion layer) were deposited using e-beam evaporation (Evaporator Evatec pro systems, Bak 501LL) and patterned using a lift-off process. The lateral walls of microchannels were 15 μm high and patterned in SU-8 using a standard photolithography process. Then, chips were partially diced using the “Chip-olate” process^56^ and sealed by lamination using a dry fil resist (DF-1050, EMS Inc., USA).

### Peripheral and smartphone application to activate the electrogate and flow monitoring

The peripheral device for monitoring the flow and activation of the electrogates has been described elsewhere^22,30^. Briefly, the peripheral comprises an Arduino microcontroller, a Bluetooth^®^ module for communicating with the smartphone, two 16-channel analog multiplexers to select the positive and the negative electrodes respectively, a 12-bit digital-to-analog convertor (DAC) to generate the electrogate voltage, an analog switch to connect the positive electrode either to the DAC output or to the flow monitoring circuit depending on the mode of operation, a 3.7 V/110 mAh Li-Po battery and its charging circuit, and a 16-contact Pogo pin connector to interface with the microfluidic chip. All components were assembled into a 3D-printed housing measuring 70 mm × 25 mm × 10 mm (see Supplementary Fig. S4). The smartphone application was developed using a Java-based platform called DroidScript. Parameters such as electrogate voltage (0 to 5 V), positive and negative electrodes (1 to 16), activation of flow monitoring or electrogating, and the activation time were manually selected using the application and sent to the peripheral via Bluetooth^®^ using the JSON data format. When the electrogating function is activated by the user, the peripheral generates the requested voltage using the DAC and applies it between the selected electrodes. When the flow monitoring function is activated, the analog switch disconnects the positive contact from the DAC and connects it to the circuit measuring the capacitance of the electrode pair for monitoring the position of the liquid. The circuit and the algorithm used for the flow monitoring has been described in detail elsewhere^30^. For the flow characterization experiments, results of the capacitance measurements were sent to the smartphone via Bluetooth^®^ and simultaneously to a PC via the USB port of the Arduino microcontroller to record the measurement data for plotting.

### Custom macro camera to take microfluidic chip photos

Photographs of microfluidic chips were taken using a Raspberry Pi Zero W with an 8-megapixel CMOS camera (camera module v2). The camera was attached to a custom XYZ stage with a tilting option. The focus and the field of view were adjusted by manually rotating the lens of the camera and changing the distance between the camera and the chip. An array of LEDs with a 0.5 mm-thick white polystyrene foil as a diffuser was used to illuminate the chip surface. The “picamera” library written in Python was used to adjust the camera settings and to take still images (e.g. see Fig. 3a).

### Tests to characterize the array

Experiments were performed under a Leica MZ16 microscope and pictures were taken using a camera Nikon 1J3. 5 µL of a blue dye was pipetted on the loading pad of a microfluidic chip. Electrogates were activated or not according to the desired flow rate, using a peripheral and a smartphone application and applying a voltage comprised between 3 and 5 V. Capacitance measurement was recorded using Realterm and curves were plotted with the software OriginLab (Origin, Version 2018, OriginLab Corporation, Northampton, MA, USA).

### Laminar flow experiments

Experiments with dye solutions were performed under a Leica MZ16 microscope and using a Nikon 1J3 camera. 5 µL of a red dye solution was pipetted on loading pad 1 and 5 μL of a blue dye solution was pipetted on loading pad 2. Experiments using fluorescein were performed using a Nikon Eclipse 90i microscope, a Nikon Digital Sight DS-1QM/H camera and a FITC filter. For this, 5 μL of fluorescein solution was pipetted on loading pad 1 and 5 μL of PBS on loading pad 2. Image processing was performed using the software Fiji and curves were plotted using the software OriginLab.

### Enzymatic assay experiments on glass slides (“off-chip”)

Assays proceeded by depositing 0.3 μL of a suspension containing microbeads functionalized with alkaline phosphatase on a microscope glass slide (76 mm × 26 mm × 1 mm, VWR, Radnor, PA, USA) followed with 0.3 μL of ELF-97 substrate (5 mM concentration). The resulting solution was covered with a thin microscope cover glass (Marienfeld, Laude-Königshofen, Germany). Images were taken under a microscope Axio Observer.Z1/7 (Zeiss, Oberkochen, Germany) and using a camera Prime – 95B (Photometrics, Tucson, AZ, USA) and a 20× objective. Bright field images were taken using a LED light source set on 4 V intensity and 10 ms exposure time. Fluorescence was monitored over time (1 picture taken every 30 s for 60 min) using a 385 nm LED-Module (1.6% light intensity), a 514/15 nm emission filter and 5 ms exposure time. The intensity of the fluorescent signal was evaluated using the mean gray value over the whole image using ImageJ. The negative control used beads without alkaline phosphatase.

### Enzymatic assay experiments on microfluidic chips

Functionalized microbeads were trapped in the bead lane of a microfluidic chip. For that, few nanoliters of 0.05% (v/v) Tween^®^ 20 surfactant in PBS were added into the loading pad servicing the bead lane to help wetting all its structures. Few hundred nanoliters of bead suspension (1% w:v) were pipetted into the loading pad for bead lane, passed the bead lane, and partially filled the capillary pump for bead lane. Beads accumulated and filled the bead lane in ~ 20 s. The suspension evaporated in ~ 40 s leaving beads packed in the bead lane. Microfluidic chips were laminated at 45 °C using the dry film resist DF-1050. 5 μL of solution containing ELF-97 (dilution × 100) was pipetted on the loading pad of the microfluidic chip, and electrogates were activated to fill the desired resistors. Experiments were visualized using a Nikon Eclipse 90i microscope, a Nikon Digital Sight DS-1QM/H camera and a ×20 objective, using a 0.5 ms exposure time for the acquisition of images in bright field. Time-lapse images (1 picture taken every 10 s for 10 min) were performed under fluorescence (80 ms exposure time, UV-2A filter) and the fluorescence intensity was evaluated in the bead lane using the software Fiji.

## Supplementary information

Programmable hydraulic resistor for microfluidic chips using electrogate arrays
