# Supplementary material for: Programmable hydraulic resistor for microfluidic chips using electrogate arrays
Source: Sci Rep. 2019 Nov 21;9:17242. doi: 10.1038/s41598-019-53885-w (PMC6872553; doi:10.1038/s41598-019-53885-w)
Supplement: Supplementary file 1 — Programmable hydraulic resistor for microfluidic chips using electrogate arrays [file 41598_2019_53885_MOESM1_ESM.docx]

Programmable hydraulic resistor for microfluidic chips using electrogate arrays

**- Supplementary Information -**

Marie L. Salva^a,b^, Yuksel Temiz^a^, Marco Rocca^b^, Yulieth C. Arango^a^, Christof M. Niemeyer^b^, Emmanuel Delamarche^a^*

(a) IBM Research – Zurich, Säumerstrasse 4, 8803 Rüschlikon, Switzerland

(b) Karlsruhe Institute of Technology (KIT) – Institute for Biological Interfaces (IBG-1), Hermann-von-Helmholtz-Platz 1, 76344 Eggenstein-Leopoldshafen, Germany

[^*^emd@zurich.ibm.com](mailto:*emd@zurich.ibm.com)

**
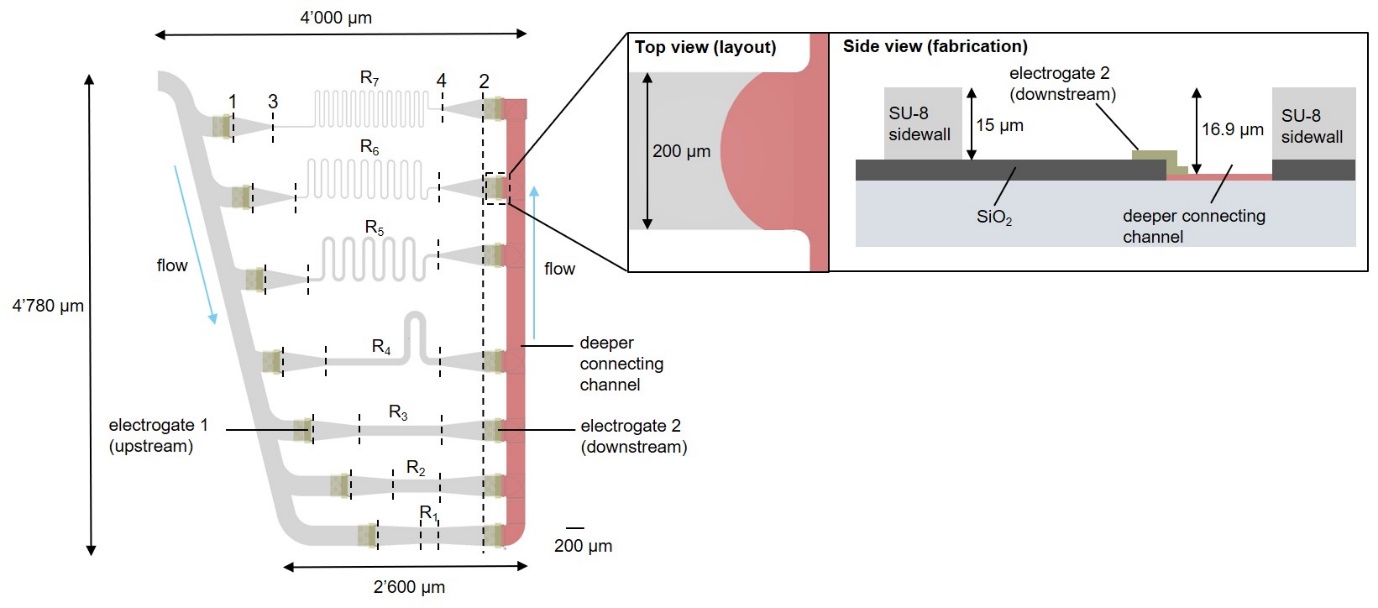
**

|  | Hydraulic resistance between positions 1 and 2 (m^-3^) | Length between positions 3 and 4 (m) | Width at positions 3 and 4 (m) | Depth (m) |
| --- | --- | --- | --- | --- |
| R_7_ | 1.39 × 10^19^ | 1.00 × 10^-2^ | 1.00 × 10^-5^ | 1.50 × 10^-5^ |
| R_6_ | 6.09 × 10^18^ | 7.50 × 10^-3^ | 1.30 × 10^-5^ | 1.50 × 10^-5^ |
| R_5_ | 9.08 × 10^17^ | 5.00 × 10^-3^ | 3.00 × 10^-5^ | 1.50 × 10^-5^ |
| R_4_ | 1.46 × 10^17^ | 2.00 × 10^-3^ | 7.00× 10^-5^ | 1.50 × 10^-5^ |
| R_3_ | 5.99 × 10^16^ | 9.00 × 10^-4^ | 1.00 × 10^-4^ | 1.50 × 10^-5^ |
| R_2_ | 3.63 × 10^16^ | 5.00 × 10^-4^ | 1.30 × 10^-4^ | 1.50 × 10^-5^ |
| R_1_ | 2.42 × 10^16^ | 2.00 × 10^-4^ | 1.60 × 10^-4^ | 1.50 × 10^-5^ |

**Supplementary Figure S1. Layout of a resistor array composed of 7 parallel resistors.** The lowest individual resistance (R_1_) is located at the “bottom” of the array and the highest one (R_7_) on “top” of the array. Electrogates 1 allow choosing which resistors to activate and one of electrogates 2 is used to let the liquid pass an individual resistor and connect with liquids present in other activated resistors. If a combination of resistors is activated, merging of liquid is performed by activating the electrogate 2 corresponding to the lowest individual resistance that is activated in order to avoid the formation of a bubble in the connecting channel, which is 16.9 μm depth. For example, if resistors 2, 4 and 6 are activated and filled, merging of liquid is performed by activating electrogate 2 of resistor 2. The insets show a top view and a side view of the intersection between electrogate 2 and the deeper connecting channel. The table gives the value and dimensions of each individual resistors calculated between positions 1 and 2, and 3 and 4.

1. **for d < w (R_1_, R_2_, R_3_, R_4_, R_5_)**

**
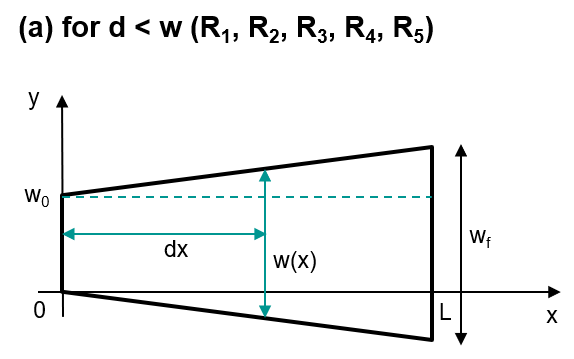
**

$$R= \int_{x = 0}^{x = L} \frac{12}{\left( 1 - 0.63 \frac{d}{w_{0}+ \frac{w_{f}-w_{0}}{L} x} \right) d^{3} \left( w_{0}+ \frac{w_{f}-w_{0}}{L} x \right)}\mathrm{dx}$$

1. **for w < d (R_6_, R_7_)**

**
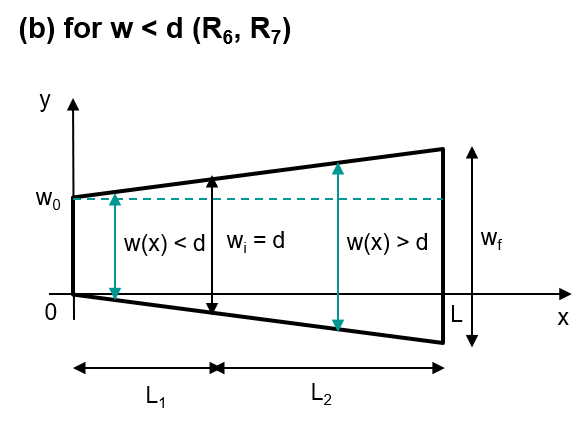
**

Determination of $L_{1}$ using Thales’s theorem $\frac{\frac{w_{f}-w_{i}}{2}}{\frac{w_{f}-w_{0}}{2}}=\frac{L_{1}}{L}$ and deduction of $L_{2}={L - L}_{1}$

$$R=\int_{x = 0}^{x =L_{1}} \frac{12}{\left( 1 -0.63 \frac{w_{0}+ \frac{w_{i}-w_{0}}{L_{1}} x}{d} \right) \left( w_{0}+ \frac{w_{i}-w_{0}}{L_{1}} x \right)^{3} d}\mathrm{dx}$$

$$+ \int_{x = 0}^{x =L_{2}} \frac{12}{\left( 1 -0.63 \frac{d}{w_{i}+ \frac{w_{f}-w_{i}}{L_{2}} x} \right) d^{3} \left( w_{i}+ \frac{w_{f}-w_{i}}{L_{2}} x \right)}\mathrm{dx}$$

**Supplementary Figure S2. Hydraulic flow resistance of a microchannel with a variable width. (a)** Sketch of a junction and corresponding mathematical equation of the hydraulic flow resistance when the depth *d* of a junction is smaller than the width *w*. This case concerns resistors R_1_, R_2_, R_3_, R_4_ and R_5_. **(b)** Sketch of a junction and corresponding mathematical equation of the hydraulic flow resistance when the width *w* of a junction is smaller than the depth *d* along a distance L_1_, and larger along a distance L_2_. This case concerns resistors R_6_ and R_7_.


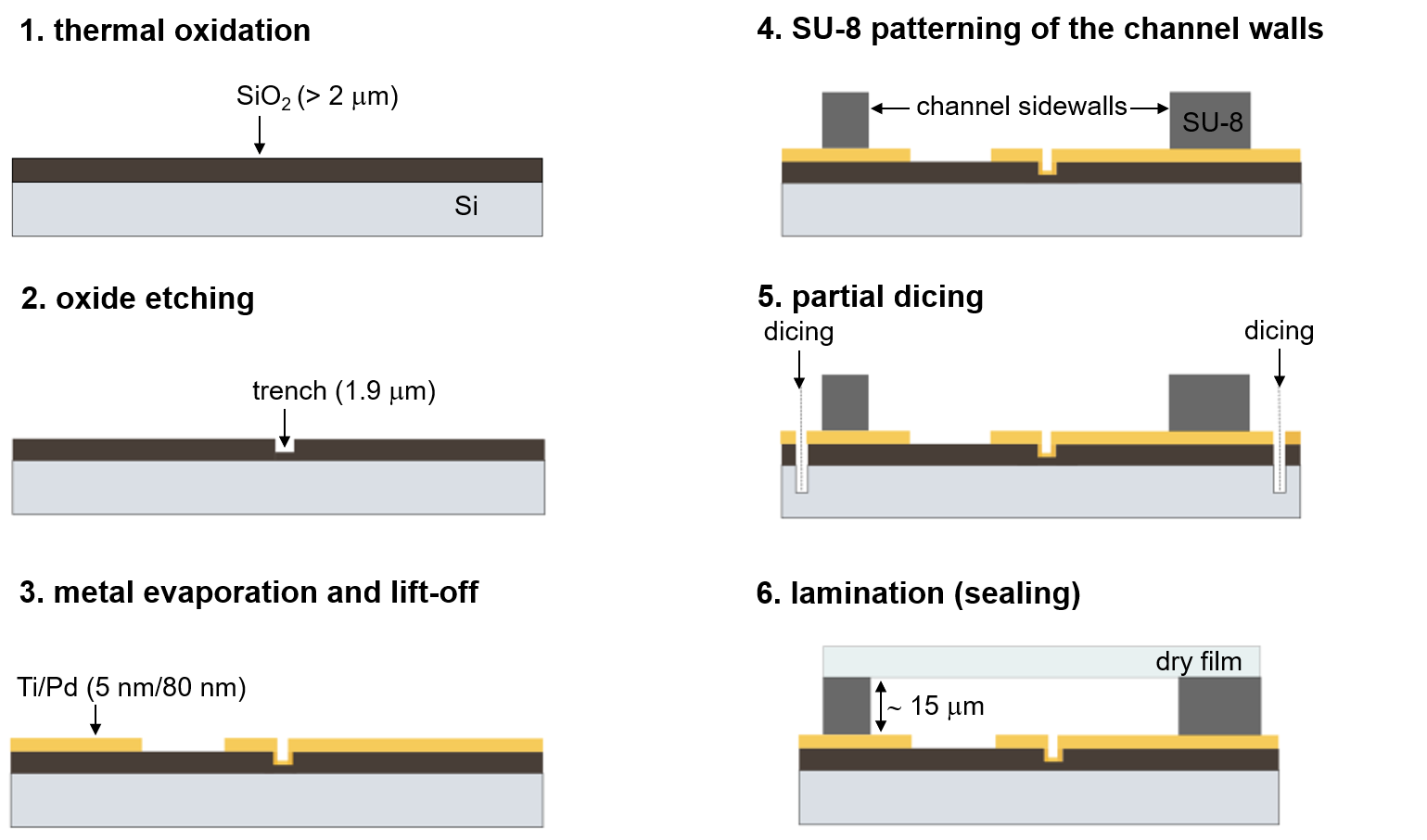


**Supplementary Figure S3. Microfluidic chip fabrication. (1)** Growing a layer (> 2 μm) of SiO_2_ on the surface of a Si wafer using thermal oxydation. **(2)** Creation of a trench (1.9 μm) by etching the oxide layer to define the pinning site of electrogates. **(3)** Deposition of a metal layer (5 nm of Ti and 80 nm of Pd) using metal evaporation and lift-off to pattern electrodes for electrogates and monitoring flow. **(4)** Patterning of SU-8 to form 15 μm sidewalls to define flow paths. **(5)** Partial dicing of the chips and **(6)** lamination with a dry film resist before experiments.


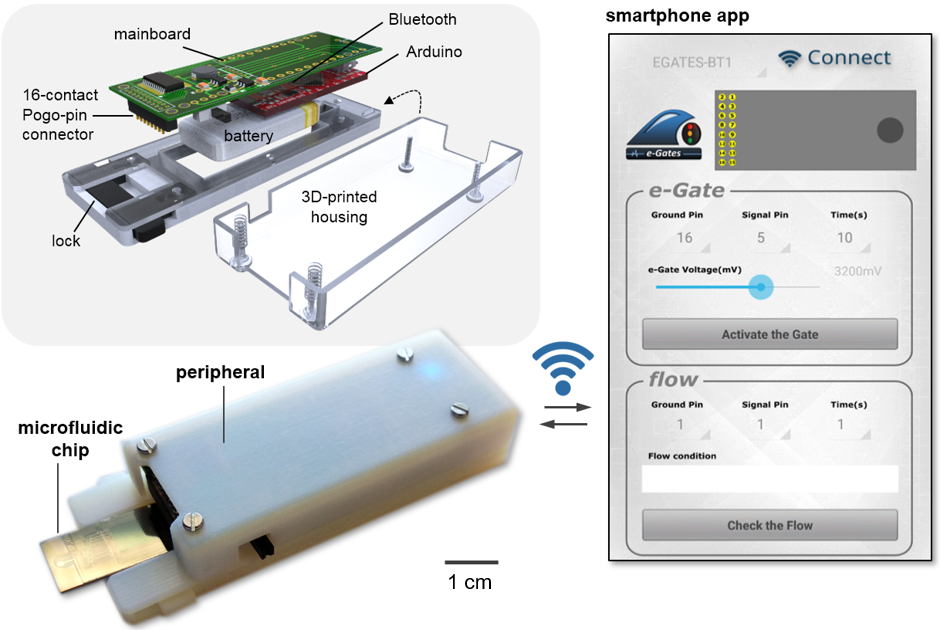


**Supplementary Figure S4. Peripheral for interfacing a microfluidic chip having a resistor array with an application on a smartphone.** Exploded view of a custom peripheral used for applying a voltage bias to select electrogates of an array upon receiving instructions from a smartphone. An application on the smartphone allows a user to select which electrogates should be activated using a specific voltage and pulse duration.

**
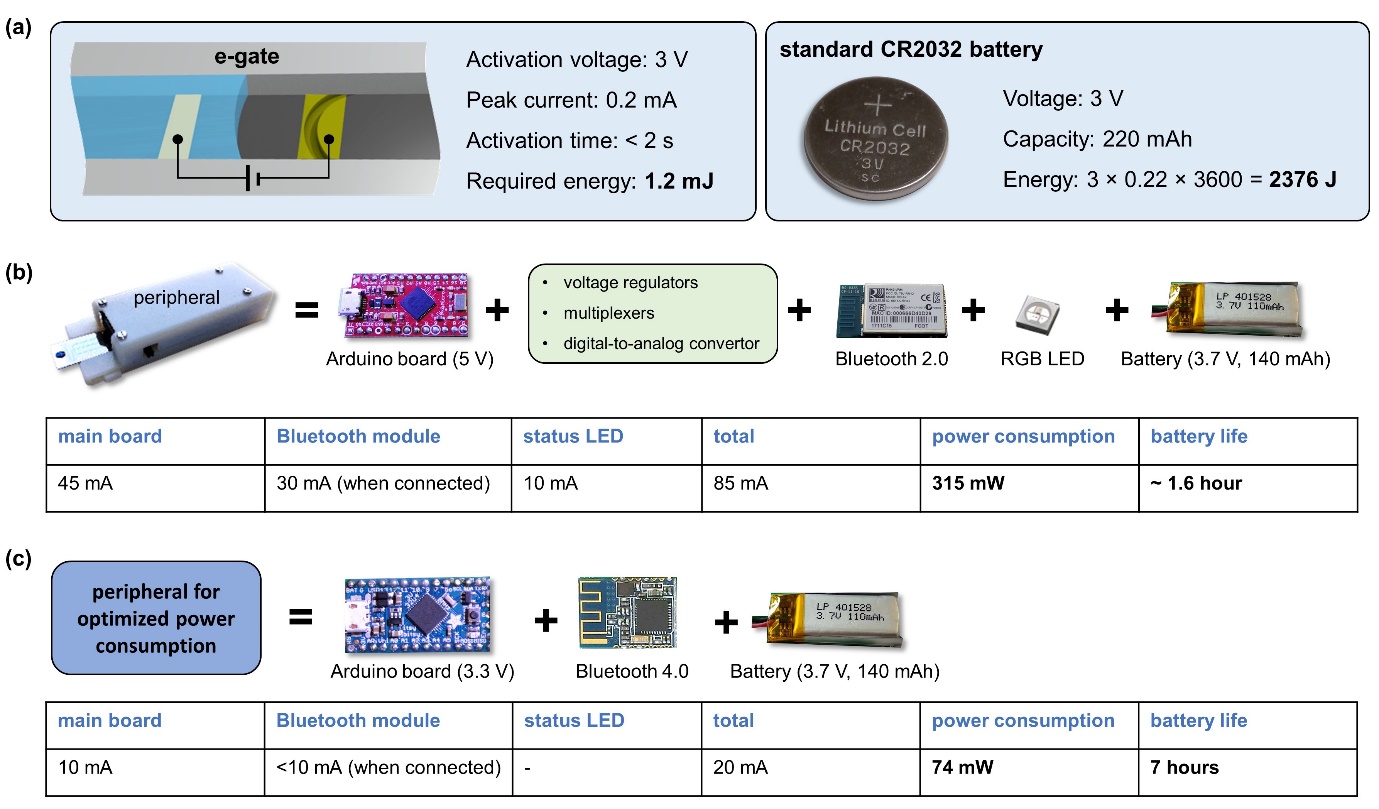
**

**Supplementary Figure S5. Power consumption for actuating electrogates. (a)** Energy required to activate an e-gate is about 1.2 mJ when 3 V activation voltage is applied for 2 s. To put this into a perspective, a standard CR2032 button cell battery could activate e-gates two million times. However, this number is not realistic because a peripheral device is required to control e-gates according to a user’s commands. **(b)** The peripheral device used in this work comprises an Arduino board, a Bluetooth 2.0 module, an RGB status LED, all powered by a compact (14 × 30 × 4 mm^3^), 3.7 V, 140 mAh Li-Po battery. There are also additional voltage regulators, multiplexers and a DAC to make this peripheral device flexible (e.g. e-gate voltage can be tuned from 0 V to 5 V and electrode pairs can be selected in any combination). Overall, the device consumes about 85 mA from 3.7 V battery, resulting in a battery life of 1.6 hour. **(c)** A peripheral optimized for power consumption would not need multiplexers and voltage regulators because 15 e-gates could be activated directly from the microcontroller by applying 3.3 V. Also, a Bluetooth 4.0 (Bluetooth Low Energy) module could be used to reduce overall power consumption during wireless communication. Such an implementation could easily boost the battery life to several hours. Power consumption could be reduced even further by applying some algorithms to put the peripheral into the “sleep” mode once all e-gates are activated and/or minimizing the use of wireless communication by reducing the sampling rate of flow monitoring.

**
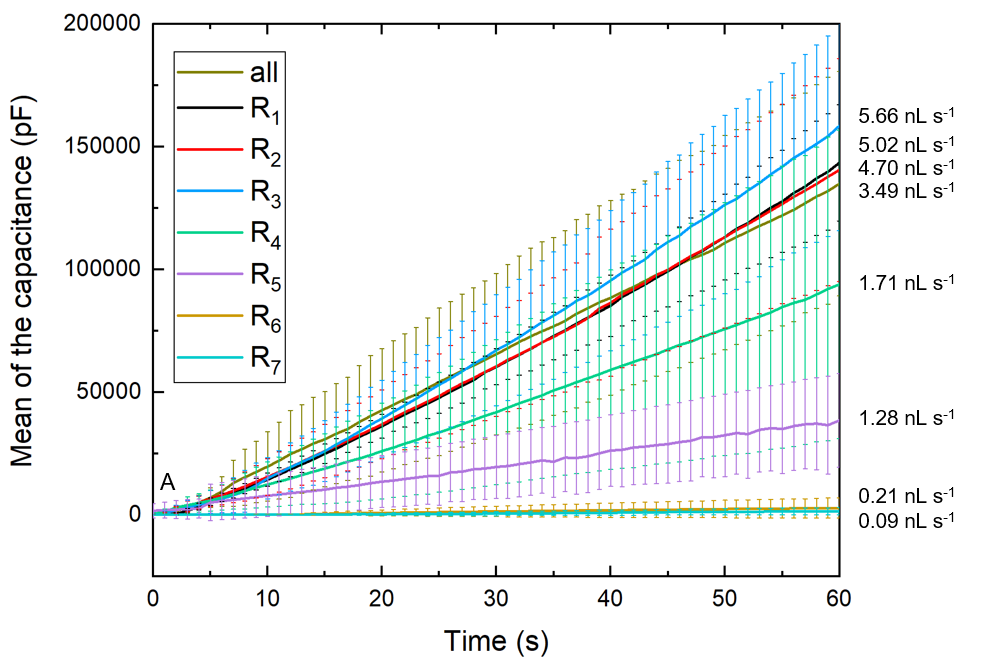
**

**Supplementary Figure S6. Flow monitoring in the capillary pump using capacitance measurements.** Graphs showing the evolution of the capacitance measured across parallel electrodes patterned in the capillary pump as a function of time for all or a single activated resistor. The graphs are an average from experiments done in triplicate and include error bars representing the standard deviations.


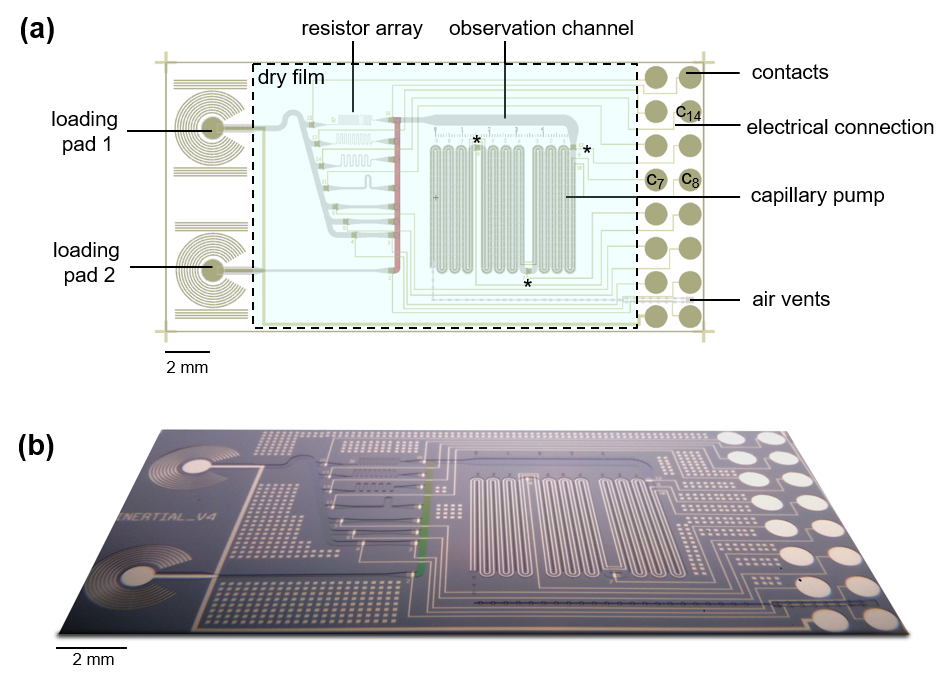


**Supplementary Figure S7. Microfluidic chip for laminar flow (a)** Microfluidic chip layout used for experiments involving laminar flow. The microfluidic chip includes two independent loading pads, a resistor array, an observation channel, a capillary pump, air vents, 16 contacts and electrical connections. Only one liquid passes through the resistor array and the liquid from loading pad 2 connects the individual outputs of each resistor. The capillary pump includes 3 electrogates (positions indicated by the stars and with corresponding contacts C_7_, C_8_, and C_14_) in order to stop the flow, for example to monitor biochemical processes in the observation channel, if desired. **(b)** Photograph of a microfluidic chip used for laminar flow experiments fabricated using 3 photolithography steps and with a flow path of 15 μm in depth.


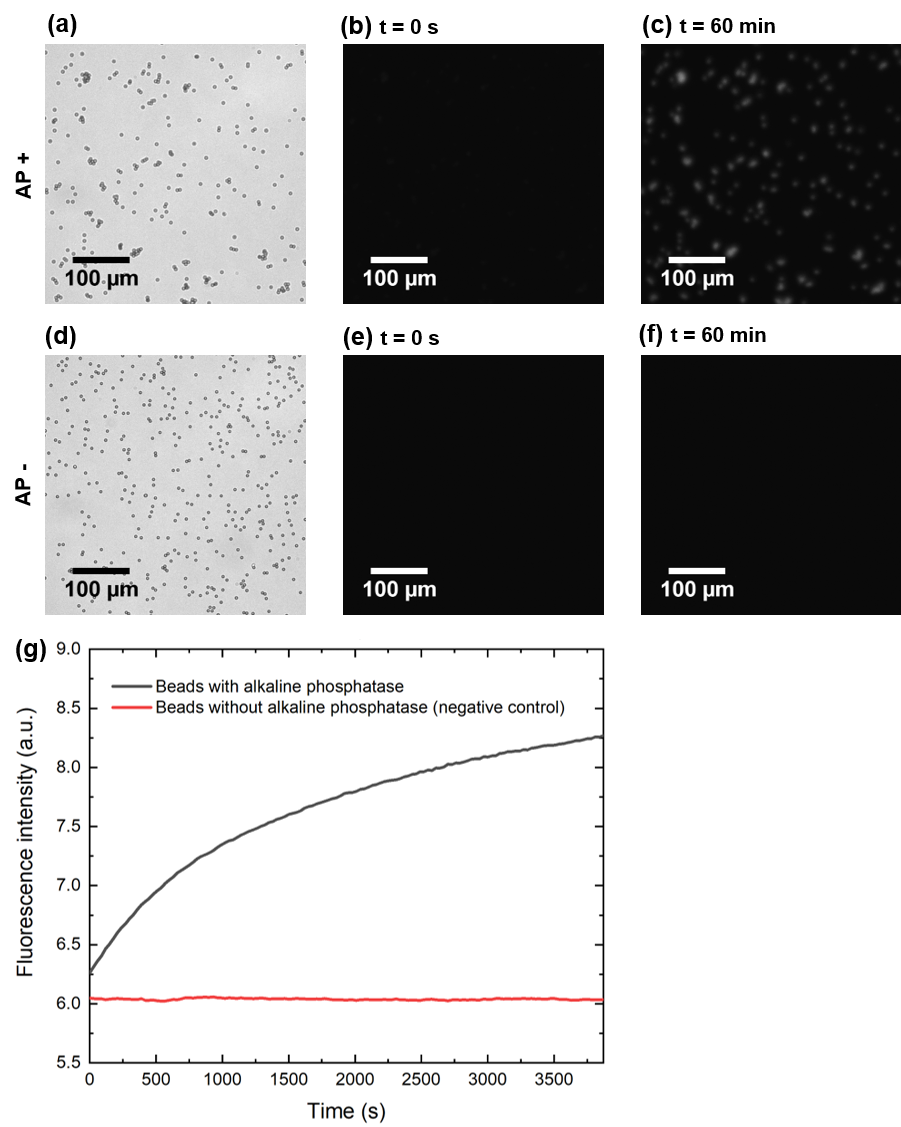


**Supplementary Figure S8. Preliminary experiments “off-chip” for enzymatic assays using alkaline phosphatase and Elf-97: fluorescent precipitating product of ELF-97 when dephosphorylated by alkaline phosphatase immobilized on 5 μm PMMA beads.** **(a)**, **(d)** Bright field images of the beads on a microscope glass slide with and without alkaline phosphatase respectively. **(b)**, **(c)** Accumulation of fluorescent product on the beads over 60 min. **(e)**, **(f)** No accumulation of fluorescent product on the beads occur without alkaline phosphatase (negative control). **(g)** Kinetics of alkaline phosphatase activity: the black curve shows an increase of fluorescence signal on the beads functionalized with alkaline phosphatase in contrast to the negative control (red curve).


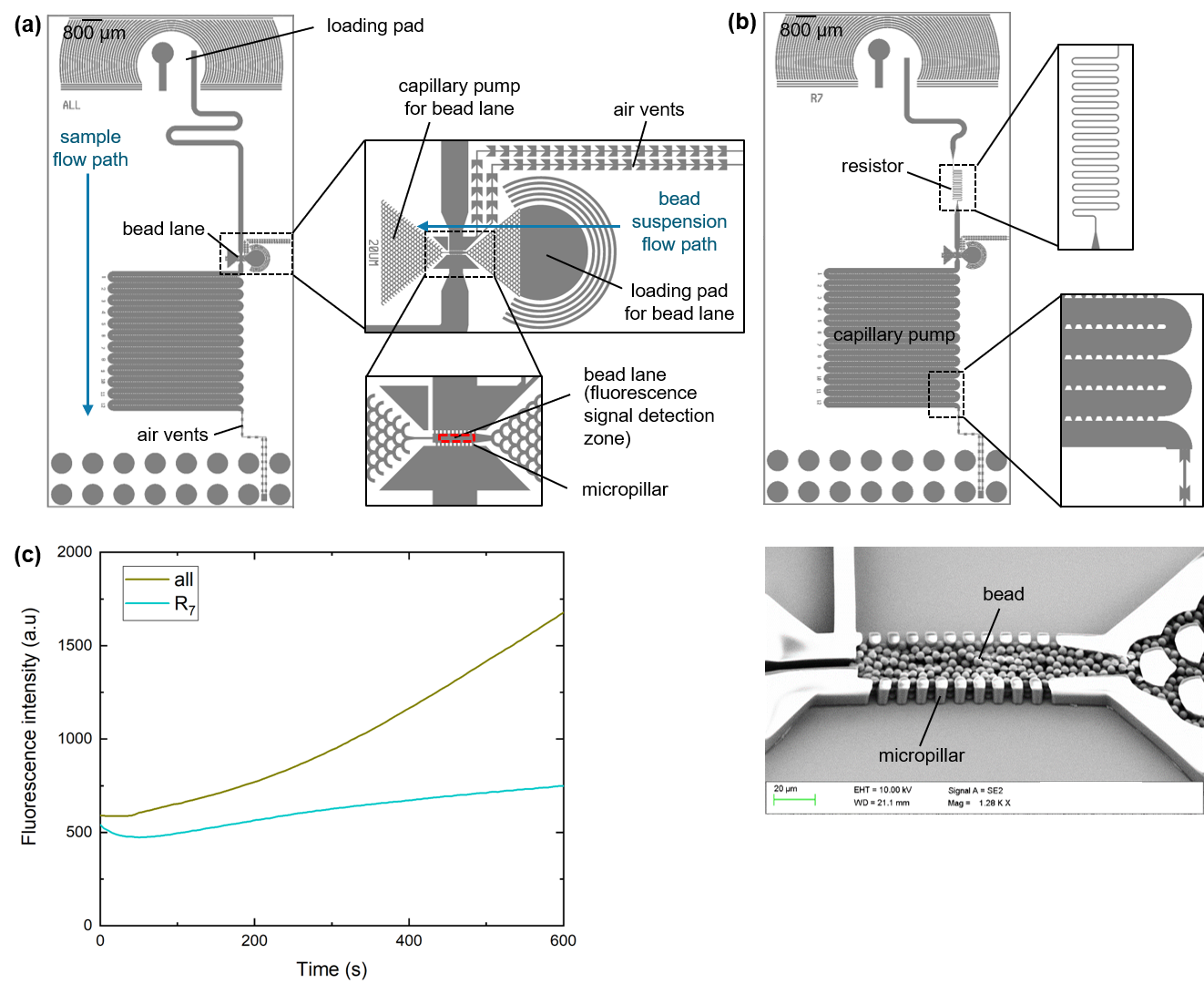


**Supplementary Figure S9. Preliminary experiments “on-chip” for enzymatic assays using alkaline phosphatase and ELF-97.** Layout of simplified (no resistor array, no electrogates) microfluidic chips designed to mimic a hydraulic resistance **(a)** corresponding to an array having all resistors activated or **(b)** only one resistor activated (e.g. R_7_, highest resistance). The main flow path of such microfluidic chips is composed of a loading pad (onto which a sample is pipetted), a hydraulic resistor, a bead lane, a capillary pump and air vents. **(c)** Graphs showing the dephosphorylation of a 0.05 mM solution of ELF-97 in PBS by alkaline phosphatase bound to microbeads immobilized in a bead lane for the microfluidic chips presented in (a) and (b). The region of interest where the fluorescence signal is measured is highlighted using a red rectangle in the inset in (a). **(d)** Scanning electron microscopy (SEM) image of a bead lane filled with 5 μm PMMA beads functionalized with alkaline phosphatase.


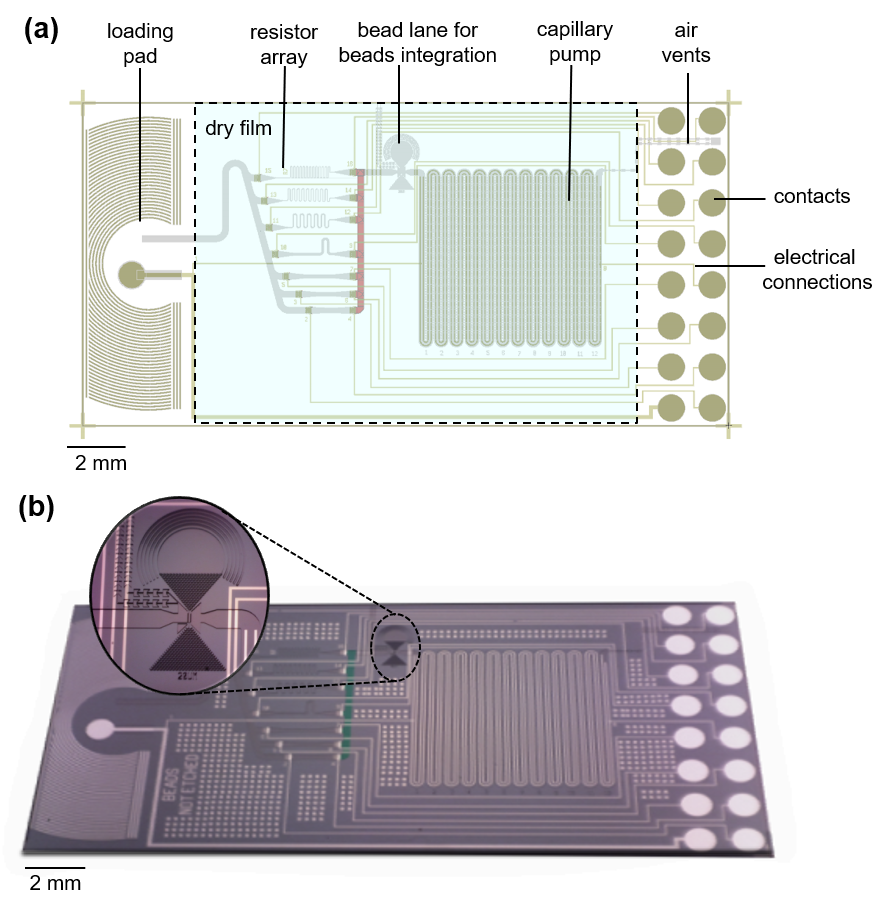


**Supplementary Figure S10. Microfluidic chip for enzymatic assays. (a)** Microfluidic chip layout used for enzymatic assay experiments. The microfluidic chip includes one loading pad, a resistor array, a bead lane structure for integrating beads coated with enzymes (here alkaline phosphatase), a capillary pump, air vents, 16 contacts, and electrical connections. **(b)** Photograph of a microfluidic chip fabricated using 3 masks and with a 15-μm-deep flow path, and inset showing the structures of a bead lane used to trap beads.
